# Cytochalasin-B-Inducible Nanovesicle Mimics of Natural Extracellular Vesicles That Are Capable of Nucleic Acid Transfer

**DOI:** 10.3390/mi10110750

**Published:** 2019-11-01

**Authors:** Anastasiya Oshchepkova, Alexandra Neumestova, Vera Matveeva, Lyudmila Artemyeva, Ksenia Morozova, Elena Kiseleva, Marina Zenkova, Valentin Vlassov

**Affiliations:** 1 Institute of Chemical Biology and Fundamental Medicine SB RAS, Novosibirsk 630090, Russia; 2 Institute of Cytology and Genetics SB RAS, Novosibirsk 630090, Russia

**Keywords:** extracellular vesicles (EVs), cytochalasin B, mesenchymal stem cells (MSCs), nucleic acid delivery, nanovesicles, freezing and thawing

## Abstract

Extracellular vesicles provide cell-to-cell communication and have great potential for use as therapeutic carriers. This study was aimed at the development of an extracellular vesicle-based system for nucleic acid delivery. Three types of nanovesicles were assayed as oligonucleotide carriers: Mesenchymal stem cell-derived extracellular vesicles and mimics prepared either by cell treatment with cytochalasin B or by vesicle generation from plasma membrane. Nanovesicles were loaded with a DNA oligonucleotide by freezing/thawing, sonication, or permeabilization with saponin. Oligonucleotide delivery was assayed using HEK293 cells. Extracellular vesicles and mimics were characterized by a similar oligonucleotide loading level but different efficiency of oligonucleotide delivery. Cytochalasin-B-inducible nanovesicles exhibited the highest level of oligonucleotide accumulation in HEK293 cells and a loading capacity of 0.44 ± 0.05 pmol/µg. The loaded oligonucleotide was mostly protected from nuclease action.

## 1. Introduction

In recent years, there has been a widespread interest in natural carriers of nucleic acids, namely, extracellular vesicles (EVs). These membrane vesicles transfer a wide variety of biologically active molecules among cells, and they are involved in different biological processes in organisms [1]. Exosomes—a fraction of EVs—are 50–150 nm vesicles produced by multivesicular bodies or from the cellular plasma membrane [2]. These EVs transfer different types of non-coding RNA or messenger RNA (mRNA) and have a specific pattern of surface proteins and lipid composition that define their tropism [3]. Exosomes are considered as potential carriers of therapeutic nucleic acids due to the fact of their small size and ability to cross different biological barriers and deliver their content to target cells [3,4,5].

The use of EVs as nucleic acid carriers is of great interest due to the lack of selective and efficient methods for in vivo nucleic acid delivery into target cells. This action is the main obstacle that prevents biomedical application of a number of potential oligonucleotide-based therapeutics and genome editing systems. Various chemical modifications, targeting techniques, and delivery systems have been developed to increase nucleic acid stability and improve targeting features [6]. Researchers have achieved limited success by conjugating oligonucleotides with lipophilic groups and ligands capable of binding to specific cellular receptors or by incorporating nucleic acids into nanoparticles composed of cationic polymers [6]. Application of some carriers, for example, liposomes or dendrimers, produced some successes as well as problems with low biocompatibility and toxicity [7,8]. Different nucleic acid modifications can alter their biological activity, subcellular localization, and cause toxic effects in vivo [9,10]. Delivery to specific cells can be achieved by using peptides or aptamers designed to bind to cell surface proteins [11,12], but the sensitivity of aptamers to nucleases or the immunogenicity of peptides restrict their application. A straightforward approach to the delivery of nucleic acids to specific cells could be the use of viral envelopes [13,14,15,16]; however, the possibilities of this approach are also limited by the immunogenicity of these carriers.

A number of studies demonstrated the ability of EVs to transfer exogenous nucleic acids, but effective methods for loading nucleic acids into vesicles have not yet been developed. Loading isolated EVs using electroporation is associated with nucleic acid aggregation [17] and is less efficient for long nucleic acids [18]. The pre-delivery of cargo into EV-secreting cells by transfection, transduction, or electroporation is limited by the influence of the transfection procedure and nucleic acids on cell survival. The use of commercial transfection agents for nucleic acid loading into isolated EVs is restricted by the impossibility of their separation [19,20]. The application of self-loading hydrophobic small interfering RNA (siRNA) analogs can alter intracellular location and cause functionality loss after delivery by EVs [21].

The use of natural EVs as delivery vectors is also complicated by laborious procedures for their isolation and purification. Therefore, attempts were made to produce artificial EV mimics. These products can be prepared by cell extrusion, covering nanoparticles with cell membrane [22], or by cell treatment with cytochalasin B [23] which causes actin filament dissociation. Shaking cytochalasin-B-treated cells causes cell disintegration and the formation of multiple vesicles that are built from cell plasma membrane. The use of EV mimics is limited by the lack of versatile and reliable methods to load them with nucleic acids, a criterion that must be rectified for their further in vivo application.

Natural EVs can be isolated either from human body fluids or cultured cell conditioned medium. In the present study, we used primary human endometrial mesenchymal stem cells (MSCs) to produce natural EVs and two types of their artificial mimics and then loaded them with synthetic single-stranded oligonucleotides. We performed a comparative analysis of natural EVs and their artificial mimics in terms of their yield, ability to be loaded with oligonucleotides, and ability to deliver their contents to human cells under different conditions.

## 2. Materials and Methods

### 2.1. Mesenchymal Stem Cell (MSC) Isolation and Characterization

Human MSCs from the female endometrial functional layer were used for EVs and their artificial mimics. The donors provided informed consent in accordance with the rules of the Local Ethics Committee. The MSCs in the third passage, isolated from pipelle biopsy diagnostic samples, were thawed according to a previously published study [24] and cultured in 75 cm^2^ culture flasks (TPP, Trasadingen, Switzerland) in Iscove’s modified Dulbecco’s medium (IMDM) (Gibco, Thermo Fisher Scientific, Waltham, MA, USA) with 10% fetal bovine serum mesenchymal stem cell-qualified (FBS MSC-qualified) (Gibco, USA), 2 mM Glutamax (Gibco, USA), and 1% antibiotic/antimycotic (Gibco, USA) in a CO_2_-incubator (Shell, Houston, TX, USA) at 37 °C, with 5% CO_2_ and under saturated humidity. The medium was changed every 3–4 days. Upon reaching a confluent monolayer, the cells were sequentially sub-cultured into 150 and 300 cm^2^ culture flasks (TPP, Trasadingen, Switzerland) using 0.25% trypsin-ethylenediaminetetraacetic acid (EDTA) solution (Gibco, USA) to obtain a suspension of single cells. Cells were reseeded every 7–14 days.

The cellular phenotype was determined according to the manufacturer’s instructions. This procedure used mouse monoclonal antibodies specific to HLA-ABC (Biolegend, Cat. 311405, phycoerythrin (PE) mouse IgG2a κ), HLA-DR (Biolegend, Cat. 307603, fluorescein isothiocyanate (FITC) mouse IgG2a κ), CD9 (Sony, Cat. 2160520, FITC mouse IgG1 κ), CD14 (Sony, Cat. 2228015, FITC mouse IgG1 κ), CD19 (Sony, Cat. 2111025, FITC mouse IgG1 κ), CD34 (Sony, Cat. 2317525, PE mouse IgG1 κ), CD45 (Sony, Cat. 2120025, FITC mouse IgG1 κ), CD73 (Biolegend, Cat. 334015, FITC mouse IgG1 κ), CD90 (Sony, Cat. 2240565, allophycocyanin (APC) mouse IgG1 κ), and CD105 (eBioscience, Cat. 12-1057-73, PE mouse IgG1 κ), all of which are antigens on the surface of human cells. The utilized isotype controls were: Sony, Cat. 2600550, FITC mouse IgG1 κ; Cat. 2600570, PE mouse IgG1 κ; Cat. 2600610, APC mouse IgG1 κ; Cat. 2601035, FITC mouse IgG2a k; Cat. 2601055, PE mouse IgG2a κ. The samples were analyzed with a NovoCyte™ flow cytometer using the included software (ACEA Biosciences. Inc., San Diego, CA, USA). To analyze in vitro MSC differentiation, cells were plated at 1 × 10^4^ cells/cm^2^, and the relevant differentiation medium was added.

Adipogenic, osteogenic, and chondrogenic differentiations were performed as previously described [25]. To induce adipogenic differentiation, cells were cultured in Dulbecco’s modified Eagle’s medium (DMEM) (Gibco, USA) with 10% fetal bovine serum (FBS) (Gibco, USA), 0.5 mM isobutyl-methylxanthine (IBMX) (Sigma, Saint Louis, MO, USA), 1 µM dexamethasone (KRKA, Novo Mesto, Slovenia), 200 µM indomethacin (Sigma, Saint Louis, MO, USA), and 1% antibiotic/antimycotic for 3 weeks. Differentiation into adipocytes was confirmed by staining differentiated cells with oil red O dye for neutral fat (Sigma, USA) as previously reported [26].

To induce bone cell differentiation, cells were cultured in DMEM with 10% FBS, 100 nM dexamethasone, 10 mM β-glycerophosphate (Sigma, USA), 50 µM ascorbate-2-phosphate (Sigma, USA), and 1% antibiotic/antimycotic. Alkaline phosphatase activity and calcium accumulation in the intercellular matrix were used as markers for osteogenic differentiation. After 14 days of cultivation in an induction medium, the alkaline phosphatase activity was examined. Calcium accumulation in the extracellular matrix was determined 21 days after induction as previously described [27]. To determine the alkaline phosphatase activity, cells were fixed with methanol and stained with nitrotetrazolium blue dye in the presence of 5-bromo-4-chloro-3-indolyl phosphate, an alkaline phosphatase substrate (Sigma, USA) as previously described [26]. To identify calcium salts in the intercellular matrix, the cells were fixed with 4% paraformaldehyde and stained with a 40 mM solution of alizarin red S (pH 4.1; Sigma, USA) as previously described [27].

To induce chondrocyte differentiation, cells were cultured in DMEM, with 1% FBS, ITS-A (Gibco, USA), 10 ng/mL transforming growth factor β1 (TGF-β1) (Sigma, USA), 50 nM ascorbate-2-phosphate, and 1% antibiotic/antimycotic. Ten microliters of the cell suspension (8 × 10^6^ cells/mL) were applied to the center of the well and incubated at 37 °C to allow cell attachment. After 2 h incubation, the chondrogenic medium was added to the well, and the cells were cultivated for 14 days in a humidified atmosphere with 5% CO_2_ at 37 °C. Chondrogenic differentiation was confirmed by accumulation of sulfated proteoglycans, acidic glycosaminoglycans, and collagen type II in the extracellular matrix as previously described [25]. Briefly, sulfated proteoglycans were determined using 1% (wt/vol) alcian blue solution (Sigma, USA) in 0.1 N HCl (pH 1.0). To identify glycosaminoglycans, 1% (wt/vol) toluidine blue solution (Sigma, USA) in 50% isopropyl alcohol was used. Type II collagen was analyzed using mouse monoclonal antibodies specific to human type II collagen (M2139; Thermo Fisher Scientific, Cat. MA1-40066). A goat antibody specific to the Fc fragment of mouse IgG molecules, conjugated with Су 3.5 (ab97036, Abcam, Cambridge, UK) were used as the secondary antibody.

Cytologic specimens stained by oil red O, nitrotetrazolium blue, alizarin red S, alcian blue or toluidine blue were visualized using an AxioScope.A1 microscope and Zen2.3 software (Zeiss, Oberkochen, Germany). To visualize type II collagen in the intercellular matrix of differentiated cells, a ZOE™ Fluorescent Cell Imager and its software (BIO-RAD, Hercules, CA, USA) were used.

### 2.2. Extracellular Vesicle (EV) Isolation

The EVs were isolated from the conditioned medium of human endometrial MSCs. Cells that reached 80–90% confluence were cultured in FBS-free medium supplemented with 0.5% human albumin for 48 h. The culture medium was harvested and centrifuged at 300× *g* for 10 min (4 °C), 2000× *g* for 15 min (4 °C), and then 12,000× *g* for 30 min (4 °C). The supernatant was collected and centrifuged at 100,000× *g* for 70 min at 4 °C (Avanti J-301, JA 30.50 Ti rotor, Beckman Coulter, Brea, CA, USA). The pellet was washed with 10 mL tris-buffered saline (TBS; 20 mM Tris-HCl, pH 7.5 and 150 mM NaCl) and subjected to ultracentrifugation overnight at 100,000× *g* (4 °C). The EV pellet was resuspended in 100 µL TBS and kept at 4 °C for one week. To check the effect of freezing on the MSC-derived EVs, the vesicles were stored at −80 °C from 48 h to 8 days. For long-term EV storage, conditioned medium was frozen and stored at −20 °C. The HepG2-derived EVs were isolated using the same protocol.

### 2.3. Determination of EV, Cytochalasin-B-Inducible Nanovesicle (CINV), and Membrane-Derived Nanovesicle (MDNV) Concentrations and Size Estimation

The concentration of isolated EVs or mimics was evaluated by measuring the total protein concentration in samples using the Qubit protein assay Kit (Thermo Fisher Scientific, USA). Prior to measurements, samples were lysed in 0.5% sodium dodecyl sulfate (SDS) for 15 min at room temperature followed by fluorescence measurement at 485/590 nm using Qubit 2.0 Fluorimeter.

Vesicle sizes were evaluated by dynamic light scattering (DLS) analysis using a Zetasizer Nano ZS (Malvern Instruments, Malvern, UK) device. Aliquots that contained 15 µg of nanovesicles were diluted with TBS, which was pre-filtered six times through a 0.22 µm filter, to 100 µL, and then the nanovesicle size was measured.

### 2.4. Detergent-Free Generation of Membrane-Derived Nanovesicles (MDNVs)

All procedures were performed on ice and under sterile conditions. To produce plasma membrane fragments, MSCs were destroyed by osmotic shock followed by mechanical fragmentation [28]. Cells were placed into 1.5 mL Eppendorf tubes using a non-enzymatic cell dissociating reagent (Versene solution) and centrifuged for 5 min at 1000× *g*. Subsequently, cells were washed with a buffer that contained 20 mM Tris-HCl (pH 6.8), 250 mM sucrose, 1 mM CaCl_2_, 1 mM MgCl_2_, 1 mM dithiothreitol (DTT), and 1 mM phenylmethylsulfonyl fluoride (PMSF). The pellet was resuspended in 1 mL of the same buffer and lysed 20 times with a 29 G needle. The solution was centrifuged at 1000× *g* for 5 min (4 °C), 15,000× *g* for 30 min (4 °C), and then ultra-centrifuged at 100,000× *g* for 90 min (4 °C). The MDNVs were resuspended in 100 µL TBS and stored at 4 °C for no longer than 48 h.

Three techniques were applied to generate MDNVs from plasma membrane fragments: (i) repeated freezing followed by thawing (Fr/Th); (ii) sonication in an ultrasonic bath (UB); and (iii) a combination of these methods. To generate MDNVs, 15 µg plasma membrane fragments, measured as total protein, were dissolved in 20 µL TBS. The Fr/Th procedure was performed by freezing samples in liquid nitrogen followed by incubation at −80 °C for 10 min. Subsequently, samples were thawed in a 25 °C water bath followed by vigorous shaking for 10 min at 700 rpm. The Fr/Th cycle was repeated three times. To generate MDNVs by sonication, a Cole–Parmer ultrasonic cleaner (model 08849-02, 220 VAC, 50 Hz, 0.5 A) was used. Two samples were simultaneously placed in the device and sonicated from 5 to 180 min at constant power. The water bath temperature was maintained at 37 ± 2 °C. When the Fr/Th and sonication procedures were combined; sonication preceded Fr/Th cycles.

### 2.5. Preparation of Cytochalasin-B-Inducible Nanovesicles (CINVs)

All procedures were performed under sterile conditions. The cytochalasin-B-inducible nanovesicles (CINVs) were prepared according to a previously described protocol [23,29] with some modifications. Briefly, human endometrial MSCs were incubated in IMDM medium that contained 10 µg/mL cytochalasin B (AppliChem GmbH, Darmstadt, Germany) for 30 min at 37 °C (with 5% CO_2_). Subsequently, cells were vigorously vortexed for 30 s and placed in 1.5 mL Eppendorf tubes. The suspension was centrifuged at 100× *g* (10 min, 4 °C), 600× *g* (20 min, 4 °C), and 15,000× *g* (30 min, 4 °C). The pellet, which contained CINVs, was washed with 1 mL TBS and resuspended in 100 µL TBS. The CINVs were stored at −80 °C until use.

### 2.6. Loading of EVs, CINVs, and MDNVs with Fluorescein (FAM)-Labelled Oligodeoxyribonucleotide

As a model for nucleic acids, we used a scrambled DNA oligonucleotide (5′-AGT-CTC-GAC-TTG-CTA-CC-3′) conjugated to fluorescein (FAM) at the 5′-terminus (FAM-ON).

Three techniques for nanovesicle loading with FAM-ON were used in this study: Fr/Th, membrane permeabilization by saponin treatment, and sonication. The Fr/Th was performed in the same manner as for MDNV generation. Sonication and saponin-mediated membrane permeabilization were performed as previously described [30] with some modifications. Sonication was performed using a Sonoplus ultrasonic homogenizer HD2070 (BANDELIN) at 20% power, with six cycles that consisted of a 4 s pulse and a 2 s pause repeated twice with cooling in an ice bath between the cycles. For membrane permeabilization, nanovesicles were treated with 0.02–0.2% saponin from *quillaja bark* (Sigma, USA) for 30 min; this treatment was accompanied by vigorous sample shaking at 700 rpm at room temperature.

Each loading experiment was repeated 2–7 times, and the total number of measurements ranged from 3 to 14.

EV loading by Fr/Th was performed in TBS, DMEM, or opti-minimum essential medium (Opti-MEM) at various FAM-ON concentrations. To obtain 0.1, 1, or 5 µM FAM-ON in the loading mixture, 0.02, 0.2, or 1 nmol FAM-ON was mixed with 15 µg nanovesicles in 200 µL TBS, DMEM, or Opti-MEM. To obtain 10, 20, or 50 µM FAM-ON in the loading mixture, 1 nmol FAM-ON was mixed with 15 µg nanovesicles in 100, 50, or 20 µL of the appropriate buffer/medium. Prior to loading in culture medium, EVs were isolated and resuspended in TBS at 5 μg/μL.

The MDNV loading with FAM-ON was performed when they were generated. Fifteen micrograms of plasma membrane fragments in 20 µL TBS or DMEM/Opti-MEM were mixed with 1 nmol FAM-ON followed by Fr/Th or sonication. When Fr/Th and sonication were combined, MDNVs were generated by sonication, and then loading with FAM-ON was performed by Fr/Th. Prior to loading in culture medium, plasma membrane fragments were isolated and resuspended in TBS at 5 μg/μL.

The CINV loading was performed as follows: 15 µg CINVs in 20 µL TBS or DMEM/Opti-MEM medium was mixed with 1 nmol FAM-ON in the same buffer/medium and subjected to Fr/Th as described above. When culture medium was used as the loading buffer, CINVs were isolated and resuspended in TBS at 5 μg/μL prior to loading.

In experiments performed with MDNVs and CINVs, the FAM-ON concentration in the loading mixture was maintained at 50 µM.

### 2.7. Flow Cytometry Analysis of EVs, CINVs, and MDNVs

Flow cytometry measurements were performed with a NovoCyte™ flow cytometer. The EVs, CINVs, and MDNVs were analyzed after absorption onto 4 µm aldehyde/sulfate latex beads (A37304, Invitrogen, Carlsbad, CA, USA) as previously described [31]. Briefly, 4 µL latex beads were added to the loading reaction mixture (see above) or 200 µL of a mixture that contained 15 µg EVs (in TBS) and incubated for 30 min at room temperature followed by incubation at 4 °C overnight with constant shaking. Subsequently, the 200 µL reaction mixture was mixed with an equal volume of 1 M glycine to block the remaining binding sites on the latex beads; the solution was incubated for 30 min at room temperature. Latex beads were precipitated by centrifugation at 4000 rpm for 5 min and then washed with 0.5% bovine serum albumin (BSA) supplemented with 10% EV-depleted FBS. The nanovesicles were either stained with monoclonal antibodies or washed twice with 200 µL TBS and analyzed by flow cytometry.

The EV surface proteins were detected as follows: Latex beads with immobilized EVs were resuspended in 200 µL 0.5% BSA and 10% EV-depleted FBS and incubated with monoclonal antibodies or isotype controls for 1 h at 4 °C. Subsequently, the latex beads were washed twice with 200 µL TBS and analyzed by flow cytometry.

Three biological markers were checked to detect endosome-specific proteins: CD9 (Sony, Cat. 2160520, FITC mouse IgG1 κ), CD63 (Sony, Cat. 2365040, APC mouse IgG1 κ), and CD81 (Sony, Cat. 2347530, PE mouse IgG1 κ). Three isotype controls (Sony, Cat. 2600550 FITC mouse IgG1 κ, Cat. 2600610 APC mouse IgG1 κ, and Cat. 2600570 PE mouse IgG1 κ) were used to measure non-specific background signals. All measurements were performed with a NovoCyte™ flow cytometer, using a 640 nm excitation wavelength for antibodies conjugated to APC (emission was 675 ± 30 nm) and 488 nm for antibodies conjugated to FITC or PE (emission was 530 ± 30 nm and 572 ± 28 nm, respectively).

At least 30,000 or more latex beads were analyzed in each experiment. A forward scatter area versus forward scatter height density plot (linear scale) was used to exclude latex bead doublets. To detect FAM-ON-loaded nanovesicles by FAM fluorescence, a 488 nm excitation wavelength was used; emission was detected at 530 ± 30 nm.

### 2.8. Cell Culture Experiments

The HEK293 cells were cultured in DMEM supplemented with 10% FBS (HyClone, USA) with 1% penicillin–streptomycin–amphotericin B (MP Biomedicals, USA) at 37 °C and 5% CO_2_. Before oligonucleotide delivery, cells were seeded in 24 well plates at 1 × 10^5^ cells/well and allowed to adhere overnight. In the morning, the medium was replaced with one of the experimental mediums (see below).

Intracellular FAM-ON delivery, mediated by EVs, MDNVs, or CINVs, was examined under three different conditions: (1) HEK 293 cells cultured in DMEM + 10% EV-depleted FBS; (2) HEK 293 cells cultured in Opti-MEM with 1 mM sodium pyruvate (Sigma, USA) and 5 mM HEPES (pH 7.3 ± 0.3, Sigma, USA); (3) HEK 293 cells cultured in Opti-MEM with 1 mM sodium pyruvate, 5 mM HEPES, and 10% EV-depleted FBS. Under each tested condition, 2–5 independent replicates were performed for HEK293 incubation with EVs, 2–4 independent replicates for MDNVs, and 2–3 independent replicates for CINVs.

Loading of 15 or 50 µg of nanovesicles was performed by Fr/Th in 20 µL DMEM or Opti-MEM (as described above). The loaded nanovesicles in the respective medium were added to the well (final volume in the well was 250 µL) and incubated for 4 or 8 h.

To estimate the level of the passive FAM-ON uptake, cells were incubated in the presence of 4 µM FAM-ON for 4 or 8 h. Intracellular delivery mediated by Lipofectamine 2000 (Invitrogen, USA) was used as a positive control. Briefly, according to the manufacturer’s recommendations, 1 µL Lipofectamine 2000 in 25 µL Opti-MEM was incubated for 5 min at room temperature. Subsequently, 0.25 nmol FAM-ON, dissolved in 25 µL Opti-MEM, was mixed with Lipofectamine 2000 (total volume was 50 µL), incubated for 20 min at room temperature, added to the cells, and incubated for 4 h. The final volume in the well was 250 µL.

After experiments, cells were fixed with 3.7% formaldehyde (Sigma, USA), washed with phosphate-buffered saline (PBS), and analyzed by flow cytometry as described above. The EV-depleted FBS was prepared by overnight ultracentrifugation (100,000× *g*, 4 °C).

### 2.9. Electron Microscopy Analysis

Negative contrast staining for transmission electron microscopy was performed according to a previously described procedure [32] with modifications. In brief, the samples were absorbed on formvar/carbon-coated and glow-discharged copper EM grids (Agar Scientific, Essex, UK) for 30 s, washed in a drop of TBS on parafilm, fixed with 0.5% glutaraldehyde for 5 min, washed in TBS, and stained for negative contrast with 0.5% uranyl acetate for 5 min. Finally, the samples were analyzed with a JEOL1400 microscope (JEOL, Tokyo, Japan) at 80 kV at the Center for Microscopy of Biological Subjects (Institute of Cytology and Genetics SB RAS, Novosibirsk, Russia).

Vesicle sizes were measured using iTEM software (Olympus Soft Imaging Solutions, Münster, Germany). The operator was blinded to the assignment of sections to experimental groups. For each sample, the measurements were performed on 40–50 randomly selected grid squares from two different EM grids. Aggregate counting was made for 20 randomly selected grid squares for each sample.

### 2.10. CINV Loading Capacity

A calibration curve was built for FAM-ON concentrations from 1 nM to 2 µM. For this purpose, 0.1–200 pmol oligonucleotide was dissolved in 100 µL TBS supplemented with 0.5% SDS, incubated for 15 min at room temperature, and the fluorescence of the solution was measured using a CLARIOStar fluorimeter (BMG Labtech, Ortenberg, Germany).

The CINVs (15 µg in 20 µL TBS) were loaded with 1 nmol FAM-ON by Fr/Th. After loading, the reaction volume was adjusted to 200 µL with TBS, and CINV-FAM-ON complexes were precipitated at 15,000× *g* (30 min, 4 °C). The pellet was resuspended in 200 µL TBS and again precipitated at 15,000× *g* (30 min, 4 °C). The resulting pellet was lysed in 100 µL TBS supplemented with 0.5% SDS (15 min at room temperature) and analyzed with a fluorimeter.

The FAM-ON Fr/Th–washing–sedimentation control (1 nmol in 20 µL TBS) was prepared similarly to CINV–FAM-ON complexes (but without vesicles).

### 2.11. Nuclease Protection Assay

Fifteen microgram CINVs were loaded with 1 nmol FAM-ON in 20 µL TBS by Fr/Th as described above. The loading mixture was diluted to 200 µL with TBS, and samples were centrifuged at 15,000× *g* (30 min, 4 °C). Treatment of CINV-FAM-ONs complexes with 10 U of mung bean nuclease (NEB) was performed in 100 µL buffer that contained 20 mM Tris-HCl (pH 7.5), 180 mM NaCl, 50 mM sodium acetate (pH 5.0), and 1 mM ZnSO_4_. The samples were incubated at 37 °C for 1 h. The reaction was stopped by the addition of EDTA (final concentration 200 mM) and ATP (final concentration 10 mM). Subsequently, 4 µL aldehyde/sulfate latex beads were added to the mixture and incubated as described above. The CINV–FAM–ON complexes incubated in the absence of mung bean nuclease were used as a positive control.

### 2.12. Statistical Analysis

Statistical analysis was performed using the non-parametric Mann–Whitney U test.

## 3. Results

### 3.1. Characterization of Human Endometrial MSCs

Mesenchymal stem cells (MSCs) are defined by the International Society for Cell Therapy as adhesive cells that carry CD105, CD73, and CD90 antigens on their surface; they are negative for HLA-DR, CD14, CD34, and CD45 antigens. These cells have the ability to differentiate directionally into adipocytes, osteocytes, and chondrocytes in vitro [33]. The MSCs from the functional layer of human endometrium (used in this work) had the following phenotype: HLA-DR^−^, CD14^−^, CD34^−^, CD45^−^, CD19^−^, CD105^+^, CD73^+^, CD90^+^, CD9^+^, and HLA-ABC^+^ (Figure S1). Furthermore, they directionally differentiated into adipocytes, osteocytes, and chondrocytes in vitro (Figure S2). Thus, these cells met the criteria established by the International Society for Cellular Therapy and were multipotent MSCs.

### 3.2. Isolation and Characterization of MSC-Derived Natural EVs and Their Artificial Mimics: CINVs and MDNVs

MSC-derived natural EVs were isolated from MSC conditioned medium using a standard centrifugation protocol (similar to what was previously described [34]). The MDNVs and CINVs were obtained from MSCs either by cell disruption followed by fragment membrane isolation (MDNVs) or by MSC incubation with cytochalasin B (CINVs). Cytochalasin B binds the fast-growing end of F-actin and blocks microfilament polymerization. This action affects cell membrane rigidity and results in the release of nano-sized vesicles (for details, see Materials and Methods).

The amount of EVs or artificial mimics in the obtained preparations was evaluated by measuring the total protein concentration. This method is fast, provides reliable data, and is often used to characterize EVs [30,35,36]. To maintain the viability of human endometrial MSCs that produce EVs, we used 0.5% human albumin. Contamination of EVs with albumin can interfere with the measurement of total protein in EV samples [37]. To test the level of potential contamination, clear, EV-free medium, supplemented with 0.5% human albumin, was subjected to the full procedure of differential centrifugation to imitate the EV isolation (see Materials and Methods). We found that up to 30% of the measured total protein in EV samples could be assigned to albumin. Since the contribution of serum proteins in isolated EV samples is usually ignored, we also present data without excluding the albumin input in the EV concentration. To exclude possible contamination of MSC-derived EV samples with serum-derived EVs, nanovesicle samples isolated from IMDM supplemented with 0.5% human albumin by differential centrifugation were stained with monoclonal antibodies to CD9, CD63, and CD81 tetraspanins. No vesicles were positive for these three markers.

We assessed the sizes of MSC-derived EVs and artificial mimics using DLS and electron microscopy [38]. Heterogeneity and morphological features of cell-derived vesicles create difficulties for the use of DLS, because DLS distinguishes vesicles that are at least 2–3 times different in size and it tends to better evaluate the size of larger particles while neglecting smaller ones. The DLS data (Table 1) showed that EV, CINV, and MDNV samples were characterized by different heterogeneity and polydispersity index (PDI), which ranged from 0.2 to 0.99 for different types of vesicles depending on the nanovesicle preparation protocol.

**Figure 1:**
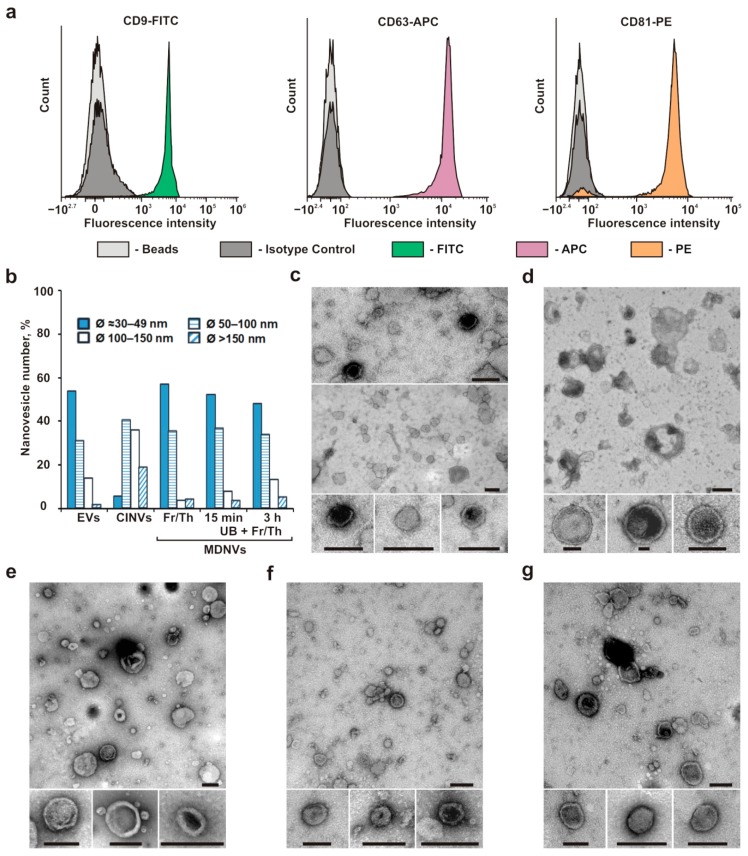
Characterization of EVs, CINVs, and MDNVs derived from human endometrial MSCs. (**a**) Natural EVs were stained with three endosome-specific tetraspanins; they exhibited the CD9^+^, CD81^+^, and CD63^+^ phenotype. (**b**) The amount of the EVs, CINVs, and MDNVs with different sizes (diameter, Ø) as evaluated by electron microscopy. (**c**–**g**) Transmission electron microscopy of (**c**) natural EVs and (**d**) CINVs isolated by 100× *g*, 600× *g*, and 15,000× *g* differential centrifugation, and MDNVs prepared by (**e**) Fr/Th, (**f**) 15 min UB + Fr/Th, or (**g**) 3 h UB + Fr/Th. Scale bar = 100 nm.

We observed that 99% of CD9^+^, CD81^+^, and CD63^+^ (Figure 1a) natural EVs, isolated from human endometrial MSC conditioned medium, using differential centrifugation, had a diameter of 210 ± 90 nm (Table 1; Figure S3), in accordance with DLS data, and 30–150 nm (*n* = 78) according to electron microscopy (Figure 1b,c). Thus, the MSC-derived EV sample consisted of small rather uniform particles (PDI = 0.2) with morphological and molecular markers similar to those of exosomes [39].

The first artificial EV mimics, CINVs, were produced from MSCs treated with cytochalasin B [23,29]. This treatment resulted in the formation of large cell fragments that were removed by sequential centrifugations at 100× *g* and 600× *g*. The CINVs were further isolated by centrifugation at 15,000× *g*. These sedimentation conditions were chosen by assuming that CINVs have a density similar to that of natural microvesicles which are produced by cells during stress and usually isolated by centrifugation at 10,000–20,000× *g*. According to DLS data, the CINV population was highly heterogeneous (Table 1, PDI = 0.5), and this data are in agreement with published results [29,40]; 75–90% of CINVs were larger than natural EVs (Table 1). However, electron microscopy analysis indicated that ~80% of CINVs were similar to the natural EV size (diameter (*Ø*) 30–150 nm), and ~20% of the nanovesicles had a diameter greater than 150 nm (*n* = 144; Figure 1b,d). It is likely that these differences can be explained by formation of CINV aggregates which can be easily seen in Supplementary Materials Figure S4. According to the electron microscopy data, 30–60% of nanovesicles in samples were in the form of aggregates (*n* = 260). For comparison, the amount of aggregates in natural EV preparations varied from 15% to 30% (*n* = 262), and these aggregates were smaller in size (Figure S4).

The MDNVs were the second type of artificial mimics investigated in this study. They were generated by detergent-free disruption of MSCs with subsequent isolation of plasma membrane fragments using differential centrifugation. The membrane fragments were used to generate MDNVs either by three Fr/Th cycles, sonication in a UB for different times (5, 15, 30, 60, 120, or 180 min), or the combination of these techniques (UB + Fr/Th). According to DLS analysis, at least 70% of MDNVs had diameters similar to natural EVs (Table 1), except for the MDNVs subjected to long ultrasound treatment (60–180 min) with or without Fr/Th. Electron microscopy evaluation of MDNV sizes was performed for samples generated by Fr/Th (Figure 1e), 15 min UB + Fr/Th (Figure 1f), or 3 h UB + Fr/Th (Figure 1g). According to electron microscopy data, approximately 50% of MDNVs had a <50 nm diameter and ~45% of vesicles were between 50–150 nm (*n* = 63–130; Figure 1b), regardless of the generation technique. Thus, ~95% of MDNVs were similar in size to natural EVs. The level of MDNV aggregation was 10–35% (*n* = 128–215) regardless of the generation technique (Figure S4). Nevertheless, formation of larger aggregates was observed in the samples subjected to prolonged ultrasound (3 h UB + Fr/Th). This observation explains the difficulties in evaluating nanovesicle sizes by DLS after the long ultrasound treatment (Table 1).

By using DLS to estimate the size of vesicles, we found 0.2–10% unstable particles with *Ø*15–60 nm (Table 1; Figure S3). At first, we suggested that these were small membrane nanovesicles recognizable by DLS, but the fact that we observed them only in the samples isolated using ultracentrifugation (i.e., EVs and MDNVs) forced us to pay closer attention to them. Electron microscopy analysis of natural EVs and three MDNV preparations (generated by Fr/Th, 15 min UB + Fr/Th, or 3 h UB + Fr/Th) revealed the presence of a small amount (less than 1%) of 30 nm non-membrane particles in these samples (Figure S5). The MSCs used in this study were obtained from clinically healthy donors at the time of biomaterial collection, but prior to collection the persons suffered from some viral infections. Thus, we believe these particles were virus-like particles, because we never detected such vesicles in EV, CINV, or MDNV preparations that originated from eukaryotic cell cultures.

The final criterion for characterizing natural EVs or mimics was the level of their production. Production of artificial nanovesicles was more efficient compared to natural EVs secreted by cells. We found that using differential centrifugation allowed the isolation of ~50 µg EVs from 50 mL conditioned medium (300 cm^2^ cell culture flask, 80–90% confluence). Artificial mimic yields, produced from the same amount of MSCs, were as high as 250–500 µg. It should be noted that the physiological features of endometrial MSC cultures permitted their use for EV, CINV, and MDNV production during passages 5–15.

### 3.3. Loading of EVs, CINVs, and MDNVs with FAM-ON

**Figure 2:**
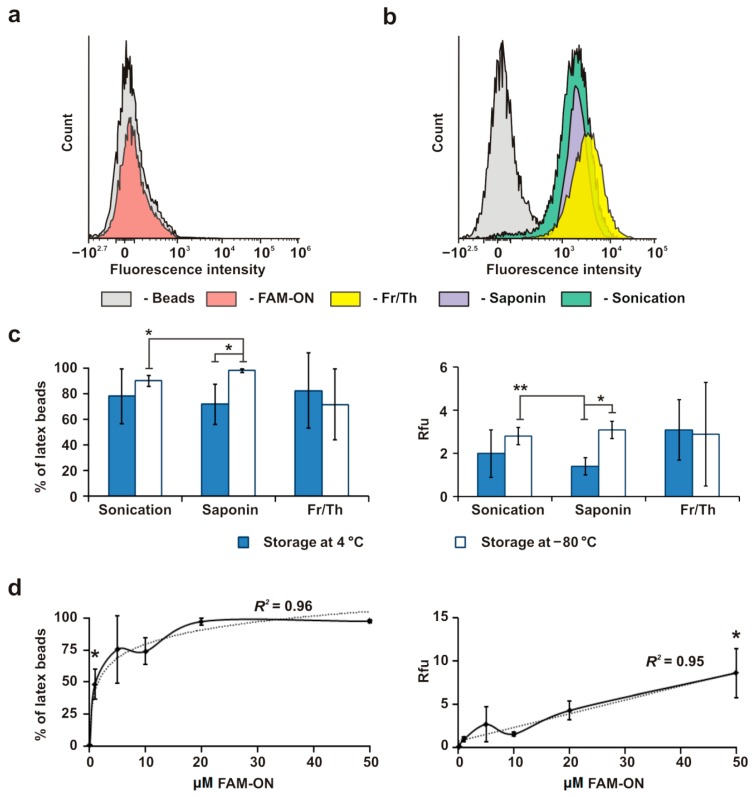
MSC-derived EV loading with FAM-ON, performed by Fr/Th, permeabilization with saponin or sonication. Flow cytometry analysis was used. (**a**) Control, non-specific binding of FAM-ON with latex beads in the absence of EVs. (**b**,**c**) Flow cytometry analysis of EV loaded under standard conditions (15 µg EVs and 1 nmol FAM-ON in 200 µL TBS) performed by Fr/Th, saponin treatment, or sonication. (**d**) Effect of FAM-ON concentration on the efficiency of EV loading by Fr/Th. Data are presented as the mean ± standard deviation; the dotted line shows the theoretical curve. All experiments were performed in TBS. Statistical analysis was performed with the Mann–Whitney U test; * *p* ≤ 0.05, ** *p* < 0.05, *** *p* ≤ 0.01.

To test the ability of EVs and the artificial nanovesicles to function as nucleic acid carriers, we loaded them with a 17-mer scrambled DNA oligonucleotide. This sequence bears a fluorescein attached via an aminohexyl linker at the 5′-terminus (FAM-ON). The use of short single-stranded DNA oligonucleotides is of great interest since antisense oligonucleotides, targeted to messenger or non-coding RNA, are widely used for regulation of gene expression [41,42]. However, the problem of their efficient delivery into target cells in vivo has not been solved yet. Since the direct detection of nanoparticles or nanovesicles using flow cytometry is complicated by their small sizes, attaching them to a detectable carrier has become a routine procedure [31]. We used 4 μm aldehyde/sulfate latex beads to bind FAM-ON-loaded EVs or mimics and analyzed these complexes by flow cytometry. We assumed that if vesicles were detected on 100% of the latex beads, an optimal level of beads saturation suitable for comparative analysis would be achieved. We used 15 µg EVs per 4 µL latex beads to analyze the CD9, CD63, and CD81 biological markers (Figure 1a) and detected signals on ~99%, ~100%, and ~92% of latex beads, respectively. Based on this data, we used 15 µg nanovesicles per 4 µL latex beads to compare different loading techniques. We verified that there was no non-specific interaction between free FAM-ON and the latex beads (Figure 2a). In each experiment, at least 30,000 events were counted; only single beads (70% of total counted beads) were used for the analysis (Figure S6).

Three approaches were tested for loading MSC-derived EVs: Fr/Th, sonication, and treatment with saponin (Figure 2b,c). Fifteen µg EVs (stored either at 4 °C no longer than 1 week or at −80 °C for 2–8 days) were mixed with 1 nmol FAM-ON in 200 µL TBS (FAM-ON final concentration was 5 μM), and EV–FAM-ON complexes were immobilized on 4 µm latex beads for flow cytometry analysis. The EV–FAM-ON complex signal was detected on 70–98% of latex beads in all experiments (Figure 2c). The relative fluorescence intensities (Rfu) that corresponded to the loading level were similar regardless of EV storage condition or loading method (Figure 2b,c). Thus, Fr/Th, sonication, and saponin provided similar efficiency in EV loading with FAM-ON.

In the oligonucleotide delivery experiments, free oligonucleotide can either be removed from the complexes or loaded nanovesicles can be used without purification from the unloaded oligonucleotide. We analyzed how pelleting EV–FAM-ON complexes by ultracentrifugation affected the overall yield of loaded nanovesicles. We observed a huge loss in nanovesicles regardless of the loading technique (Figure S7). Therefore, in our experiments we used loading mixtures without any fractionation and controlled the level of FAM-ON self-penetration into the cells.

The dependence of EV loading efficiency on the FAM-ON concentration in the loading mixture is shown in Figure 2d. In these experiments, EVs were loaded with FAM-ON by Fr/Th. As the FAM-ON concentration increased, the loading level reached a plateau (95–99% of FAM-positive beads at 20 µM), but the particle Rfu, which corresponded to the amount of loaded FAM-ON, linearly increased within the entire concentration range and exhibited no tendency to plateau. Given that the dependence of the loading efficiency on the oligonucleotide concentration was linear, we used 50 µM FAM-ON to load nanovesicles in future experiments.

**Figure 3:**
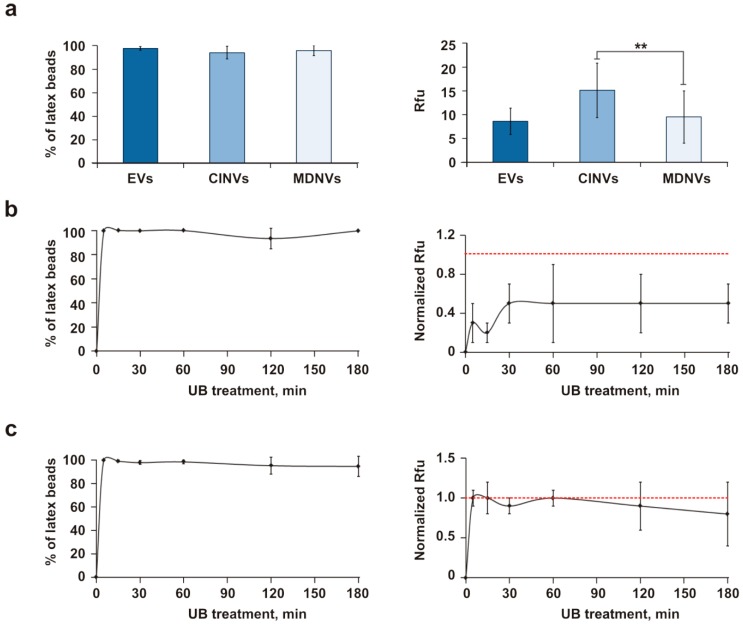
Loading of CINVs and MDNVs with FAM-ON. (**a**) Comparison of loading EVs, CINVs, or MDNVs with FAM-ON by Fr/Th. (**b**) Generation of MDNVs by UB in solution that contained FAM-ON. (**c**) Generation of MDNVs by UB followed by loading with FAM-ON by Fr/Th. The EV–FAM-ON, CINV–FAM-ON, and MDNV–FAM-ON complexes were immobilized on 4 µm aldehyde/sulfate latex beads and analyzed by flow cytometry. To correctly compare different MDNV generation techniques, Fr/Th was chosen as a positive control, and the observed Fr/Th Rfu level was set at 1 (shown as the red, dashed line). All experiments were performed in TBS that contained 15 µg nanovesicles and 50 µM FAM-ON. Data are presented as mean ± standard deviation. Statistical analysis was performed with the Mann–Whitney test; * *p* ≤ 0.05, ** *p* < 0.05, *** *p* ≤ 0.01.

The next step of this study was devoted to examining whether the nanovesicle mimics would be loaded with oligonucleotide as efficiently as natural EVs. Based on previous observations, 15 µg nanovesicle mimics were loaded with 1 nmol FAM-ON in 20 µL TBS under different conditions. The MDNV loading was performed using three techniques: MDNVs were generated in solution, which contained FAM-ON, using (i) sonication (UB), (ii) Fr/Th, or (iii) a combination of UB and Fr/Th; CINVs were loaded with FAM-ON by Fr/Th. Overall, loading mimics by Fr/Th occurred with similar efficiency to natural EVs (Figure 3a). Application of UB for MDNV loading produced poorer loading when compared to Fr/Th (Figure 3b), while the UB and Fr/Th combination provided similar loading to Fr/Th alone (Figure 3c). Thus, EVs and the artificial mimics were loaded with oligonucleotide by Fr/Th with similar efficiency.

The TBS buffer used for nanovesicle loading did not work well with cell experiments. Thus, we compared loading efficiency of nanovesicles in TBS with that achieved in DMEM and Opti-MEM (Figure S8). We found that nanovesicle loading with FAM-ON was not affected by the utilized buffer/medium; DMEM, Opti-MEM, and TBS were suitable to prepare nanovesicle-FAM-ON complexes.

### 3.4. FAM-ON Delivery into HEK293 Cells Mediated by EVs, CINVs, and MDNVs

**Figure 4:**
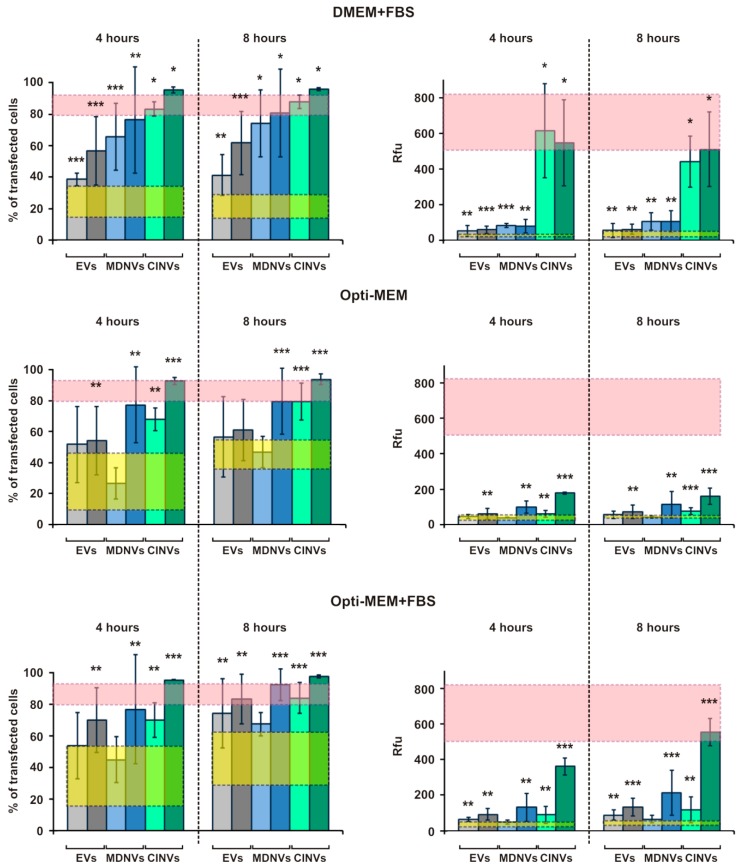
FAM-ON delivery into HEK293 cells mediated by EVs, CINVs, and MDNVs. Fifteen and 50 µg EVs, CINVs, or MDNVs were loaded with FAM-ON (50 µM) by Fr/Th. The complexes were used for FAM-ON delivery into HEK293 in DMEM + 10% FBS, Opti-MEM, or Opti-MEM + 10% FBS for 4 or 8 h. Lipofection was performed corresponding to the manufacturer’s recommendations (1 µL Lipofectamine 2000, 0.25 nmol FAM-ON per 10^5^ cells, final volume = 250 µL). Data are presented as the mean ± standard deviation. FAM-ON control and delivery of FAM-ON using Lipofectamine 2000 are indicated as yellow and pink box, respectively (± SD region). The experiments are indicated in the following colors: EV–FAM-ON—gray, MDNV–FAM-ON—blue, CINV–FAM-ON—green, 15 µg—light colors, 50 µg—dark colors. Statistical analysis was performed with the Mann–Whitney U test and was done in comparison with FAM-ON control; * *p* ≤ 0.05, ** *p* < 0.05, *** *p* ≤ 0.01.

The ability of EVs, CINVs, and MDNVs to transfer the FAM-ON into cells was tested using HEK293 cells. Three slightly different conditions were applied to study the influence of cell medium compositions on the efficiency of nanovesicle-mediated intracellular FAM-ON accumulation: (i) oligonucleotide delivery in DMEM supplemented with 10% EV-depleted FBS; (ii) oligonucleotide delivery in Opti-MEM supplemented with 1 mM sodium pyruvate and 5 mM HEPES (pH 7.3); (iii) oligonucleotide delivery using the conditions in (ii) but supplemented with 10% EV-depleted FBS. In these experiments, 15 or 50 µg EVs, CIMVs, or MDMVs loaded with FAM-ON (50 µM in the loading mixture) by Fr/Th in the appropriate culture medium were added to HEK293 cells followed by cell incubation for 4 or 8 h. The final FAM-ON concentration in the well was 4 µM. The level of FAM-ON self-penetration was estimated in control experiments, where the cells were incubated for the same time in the presence of 4 µM FAM-ON without carriers (Figure 4, FAM-ON control). The level of intracellular Lipofectamine-2000-mediated FAM-ON accumulation was used as a positive control (for details, see Materials and Methods). After incubation, the cells were washed with PBS, fixed with 3.7% formaldehyde, washed again with PBS, and analyzed by flow cytometry.

Data on intracellular nanovesicle-mediated FAM-ON accumulation are summarized in Figure 4. For the three types of nanovesicles used under the three different conditions, the percentage of FAM-positive cells in the population and Rfu are shown. Analysis of intracellular nanovesicle-mediated FAM-ON accumulation clearly demonstrated that the highest and most substantial levels of oligonucleotide delivery were achieved only when 50 µg FAM-ON-loaded EVs, MDMVs or CIMVs were used. Efficiency of oligonucleotide delivery increased from EVs ≤ MDMV < CIMV (for 50 µg) and did not change significantly depending on the exposure time (compare data for 4 and 8 h; Figure 4). The only exception was CIMVs and Opti-MEM + FBS, where there was a 30% Rfu increase observed after 8 h compared to 4 h incubation. Thus, 4 h incubation was sufficient to transfer FAM-ON complexed with nanovesicles into the cells. It is worth noting that interpretation of the MDNV–FAM-ON data is complicated due to the high variability. Apparently, the ability of MDNVs to deliver nucleic acids can depend on the stability of FAM-ON-loaded MDMVs and strongly depends on environmental conditions.

The similar oligonucleotide delivery efficiencies of EV–FAM-ON (50 µg) under the three conditions indicated that the medium and FBS presence did not influence EV-mediated oligonucleotide delivery. However, for CINVs, the presence of EV-depleted FBS in the medium stimulated FAM-ON intracellular accumulation; DMEM + FBS provided higher levels of oligonucleotide delivery compared with Opti-MEM + FBS (Figure 4, green bars). Interestingly, in the serum-free conditions, the HEK293 Rfu, but not the number of FAM-positive cells, decreased significantly, data that indicate less efficient interaction between FAM-ON-loaded CINVs and cells.

Under optimal conditions (15 or 50 µg FAM-ON-loaded CINVs; DMEM + FBS, 4 h), CINVs were the most effective as delivery vectors among the tested nanovesicles. They provided intracellular FAM-ON accumulation that was almost as high as that achieved with Lipofectamine 2000: ~85% of FAM-positive cells and 660 ± 150 Rfu (Figure 4, pink box).

Notably, artificial mimics derived from plasma membrane can differ from EVs with respect to the amount and nature of surface proteins. It is possible that the observed oligonucleotide delivery efficiency differences between the mimics and natural EVs could be related to the method used to estimate the nanovesicle quantity by measuring the total protein concentration in the samples.

### 3.5. Evaluation of Fr/Th CINV Loading Capacity with FAM-ON

Finally, we estimated the amount of FAM-ON loaded to CINVs, which provided efficient FAM-ON accumulation in HEK293 cells. In these experiments, 15 µg CINVs were loaded with FAM-ON (50 µM in the loading mixture) by Fr/Th. The calibration curve was built by measuring fluorescence of FAM-ON solutions that contained from 0.1 to 200 pmol of oligonucleotide in 100 µL TBS supplemented with 0.5% SDS.

**Figure 5:**
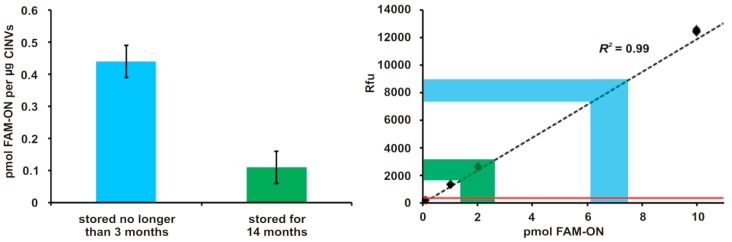
Analysis of CINV loading capacity. The quantity of FAM-ON, which were loaded to 15 µg CINVs by Fr/Th, measured by relative level of fluorescence intensity. Nanovesicles, which were stored at −80 °C no longer than 3 months, are shown in blue color, and those stored for 14 months are shown in green. The calibration curve is shown by the dashed line. The FAM-ON Fr/Th–washing–sedimentation control is shown by the red, solid line.

To evaluate the amount of FAM-ON bound/loaded on/in CINVs, FAM-ON-loaded nanovesicles were pelleted by centrifugation at 15,000× *g*, washed with TBS, lysed in TBS supplemented with 0.5% SDS, and sample fluorescence was measured. Control of unspecific FAM-ON absorption in the tube during Fr/Th and sedimentation procedure (Figure 5, red, solid line FAM-ON Fr/Th–washing–sedimentation control) showed negligible residual fluorescence (Rfu TBS + 0.5% SDS or empty well was ≈70 Rfu, FAM-ON Fr/Th–washing–sedimentation control was ≈400 Rfu).

Fifteen micrograms of CINVs contained at least 6.6 ± 0.7 pmol FAM-ON (Figure 5, blue color), which corresponds to 0.44 ± 0.05 pmol FAM-ON per 1 µg CINVs. Analysis of the effect of CINV storage on the nanovesicle loading efficiency revealed that CINVs stored at −80 °C for 14 months were four times less efficiently loaded with FAM-ON (0.11 ± 0.05 pmol FAM-ON/µg CINVs) compared to the nanovesicles stored at −80 °C for no longer than 3 months.

To determine whether the oligonucleotide was protected from nucleases after loading into CINVs, CINV-FAM-ON samples were treated with mung bean nuclease which exhibits 3′- and 5′- exonuclease activity with respect to single-stranded DNA/RNA. The FAM-ON loaded into CINVs was protected from cleavage, data that indicate FAM-ON was mostly located inside the nanovesicles. Specifically, after treatment with mung bean nuclease, the relative level of fluorescence was decreased to 60%, while the percentage of FAM-positive latex beads remained at the same level (90%).

## 4. Discussion

Studies in recent years demonstrated the potential of EVs as vectors for the delivery of therapeutics in different cells. However, a number of hurdles must be overcome to develop EV-based carriers of therapeutic value: Methods of efficient EV production remain to be developed, and efficient EVs loading techniques should be elaborated. Differential centrifugation still remains the most used EV isolation method [43,44]. This method neither affects EV ultrastructure nor introduces various contaminations to the EV samples [45]. However, it is laborious and difficult to scale.

In this study, we isolated EVs from the conditioned medium of human endometrial MSCs; these EVs demonstrated promising potential in cardiovascular therapy [46]. As a component of the stroma, MSCs are crucial for creating an appropriate microenvironment which is necessary for maturation and differentiation of the stem cells and plays the role of linker for control and regulation between the cell and the whole organism [47]. Important MSC biological features include immunomodulating properties; directing migration to sites of inflammation, reparation, and regeneration [48]; and secretion of biologically active substances such as interleukins, chemokines, growth factors, inhibitors of type 1 and type 2 metalloproteinases, extracellular matrix molecules, etc., and exosomes that contain proteins and micro-RNA which can be used to regulate signaling pathways in other cells.

We estimated that the EV yield from human endometrial MSCs was approximately 50 µg per 50 mL conditioned medium (300 cm^2^ culture flask), a value that is comparable with high EV yields obtained for embryonic stem cell (ESC)-MSCs or fetal-MSCs [49]. EV implementation in extensive research is complicated by their laborious isolation procedure. Moreover, the use of primary cell culture for EV secretion creates additional restrictions for the production of large nanovesicle quantities. For effective EV secretion, human endometrial MSCs should be in close contact (80–90% confluence). Thus, the amount of biomaterial and the rate of cell division determine the EV yield. Moreover, EV secretion by human endometrial MSCs was limited to 15th passage, after which time it almost completely stopped.

Another difficulty of EV application for therapeutic purposes is associated with the possibility of EV sample contamination with non-vesicle structures. The presence of proteins, lipoproteins, and non-characterized particles in EV samples obtained from biological body fluids or primary cell culture medium is a common problem [50]. In MSC-derived EV samples, we found up to 1% of 30 nm non-membrane particles that had a different morphology from EVs. Some viral particles have similar sizes to exosomes and can be co-purified with them by ultracentrifugation [51]. Some viruses may be enclosed in a membrane, and thus they can resemble exosomes [52]. Human immunodeficiency virus type 1 (HIV-1) particles have a density similar to that of exosomes (≈1.1–1.2 g/mL), and application of gradient density centrifugation for their separation is not effective. Among the known approaches for vesicle isolation, the separation of viral particles and EVs using specific antibodies can be considered as a promising tool. However, a recent report demonstrated that the application of antibody-coated magnetic beads for EV isolation changes EV morphology (compared to EVs isolated by differential centrifugation) [53]. Thus, methods that allow the production of preparative amounts of EVs suitable for clinical use have not yet been developed.

The potential for EVs to transfer exogenous nucleic acids has been unambiguously demonstrated in this and previous studies [18,54,55,56,57]. In our study, we used three previously reported techniques [30] to load MSC-derived EVs with FAM-ON. We showed that Fr/Th, sonication, and saponin treatment provided similar EV loading levels (Figure 2c).

The issue of EV storage requires special discussion. Four-day storage of EVs at −80 °C increased their aggregation in comparison to freshly isolated vesicles or those stored at 4 °C [58]. Mouse bone marrow MSC-derived EVs, stored at −80 °C, lost their functionality [59]. We did not observe negative effects of EVs storage at −80 °C on loading capacity, excluding the increased data variability after Fr/Th. These results neither confirm nor refute the reinforcing effect of EV storage at −80 °C on their aggregation. Apparently, aggregation was not extensive enough to significantly decrease loading efficiency. We observed aggregation of EVs derived from different cell types, which ranged from 10–30% regardless of the EV storage conditions (data not shown). We did not observe differences in EV loading efficiency regardless of loading techniques and nanovesicle storage conditions. However, taking into account the mentioned reports, we did not use EVs stored at −80 °C in HEK293 experiments.

We selected the Fr/Th procedure as the most convenient technique for nucleic acid loading into EVs. It allowed us to perform experiments under sterile conditions and excluded uncontrolled heating of the samples (which typically occurs during sonication) and the problem of biocompatibility that accompanies the use of saponin. Although Haney with co-authors [30] demonstrated the advantage of sonication for loading catalase into EVs, the use of this approach for nucleic acids is limited by degradation and aggregation issues [57]. We found that the loading efficiency of MSC-derived EVs with FAM-ON, performed by Fr/Th, linearly increased as the oligonucleotide concentration in the loading mixture increased (Figure 2d). The maximum tested FAM-ON concentration (50 µM) was used to load EVs for oligonucleotide delivery into HEK293 cells. Under the utilized conditions, we observed relatively low FAM-ON accumulation in HEK293 cells mediated by EVs (Figure 4). We speculate that this poor level of oligonucleotide delivery occurred due to the fact of an inadequate combination of EV donor and acceptor cells. The absence of significant changes with an approximately three-fold increase in EV concentration suggests low affinity of the MSC-derived EVs to HEK293 cells rather than potential poor oligonucleotide delivery ability.

The artificial mimics of natural EVs studied in this work are characterized by the following advantages: (i) simple isolation procedure, (ii) high yield, (iii) membrane organization, and (iv) sizes similar to EVs. Our results showed that mimic generation produced up to 5–10 fold more nanovesicles compared to the amount of EVs secreted by the same number of cells. The use of the cells (but not conditioned medium) for nanovesicle preparation simplified their isolation procedure and solved the problem of sample contamination with serum proteins.

Most of the artificial nanovesicles exhibited sizes similar to natural EVs. Indeed, 80% and 95% of CINVs and MDNVs, respectively, were similar in size to EVs. Smaller particles can better cross biological barriers. The use of 15,000× *g* sedimentation for CINV isolation produced nanovesicles from *Ø*30–150 nm. In other studies, CINVs were isolated by a similar procedure [60,61], but the produced nanovesicle samples were not fully characterized. Application of a lower sedimentation speed leads to the isolation of larger CINVs. For example, SH-SY5Y-derived CINVs, sedimented at 2000× *g*, contained up to 15% of nanovesicles that had a diameter ≤ 200 nm [29]; this value was less than 5% for adipose-tissue-derived MSC CINVs [62].

The CINVs significantly differed from EVs and MDNVs. They were obtained by specific disruption of the cell inner structure by a chemical agent, namely, cytochalasin B. Cytochalasin-B-inducible nanovesicles (CINVs) can mimic activities of parental cells [60,61,62], data that suggest their applicability for cell-free therapy. Cytochalasin-B-inducible nanovesicles can fuse with the membrane of recipient cells [62], similar to EVs. Indeed, we demonstrated that CINVs can be loaded with DNA oligonucleotide and effectively deliver cargo into human cells in the presence of FBS. The presence of serum proteins in the medium probably ensured sufficient nanovesicle stability during in vitro experiments with cells, similar to maintaining the viability of MSCs. We think that the potential of CINVs was not fully revealed by us due to the high level of their aggregation (30–60%). Recently, it was reported that application of a buffer, which contains serum for CINV storage, stabilizes their dispersed form, in contrast to serum-free buffer [63]. To exclude possible encapsulation of serum proteins into CINVs along with FAM-ON, we did not use this approach in this study.

In our experiments, we achieved a loading efficiency of 0.44 ± 0.05 pmol FAM-ON per 1 µg CINVs. Furthermore, at least 60% of the loaded oligonucleotide was protected from nuclease action. The level of CINV loading with FAM-ON was similar to the level of cholesterol-modified siRNA binding with EVs [21], results that indicate high loading efficiency. Long-term CINV storage at −80 °C decreased their capacity to be loaded by a factor of four, data that can be explained by increased nanovesicle aggregation.

There were similar loading efficiencies for both mimics and natural EVs. Nonetheless, only CINVs exhibited high oligonucleotide delivery efficiency close to that achieved with cationic liposomes. It is clear that the CINV action was not merely mediated by the protein surface composition, because the CINVs and MDNVs had similar surface proteins. The visible effect of cytochalasin B on actin filaments is detected for concentrations 100 times lower than usually used to destroy actin [64]. We do not know how much cytochalasin B remained in nanovesicles after CINV isolation and washing. It is unclear how cytochalasin B influenced CINV fine structure and behavior. It is possible that this actin-destabilizing agent created a specific ultrastructure for CINVs that mediated the effective vesicle–cell interaction.

In light of this work, the composition and internal content of the CINVs and the mechanism of their interaction with the cells should be studied in greater detail. Whether the CINV action is selective and the role that nanovesicle surface receptors play in the interaction with cells remains to be investigated. We think that the structure and behavior of CINVs deserves a more detailed study, including estimating the amount of toxin in the nanovesicles and its effect on cell–vesicle interactions.

## 5. Conclusions

In this study, we investigated human endometrial MSC-derived natural EVs and two types of their artificial mimics as vectors for delivery of oligonucleotides into human cells. The nanovesicles demonstrated different potentials for intracellular delivery of DNA oligonucleotide; CINVs were the most promising oligonucleotide carriers, denoted by the highest efficiency of the oligonucleotide delivery stimulated by the presence of serum. The Fr/Th allowed loading of approximately 0.44 ± 0.05 pmol FAM-ON per 1 µg CINVs, and the loaded oligonucleotide was protected from the action of nucleases.

## Figures and Tables

**Table 1:** Size distribution of MSC-derived EVs, CINVs, and MDNVs, as evaluated by DLS.

Type of Nanovesicles	*Ø* ≤ 50 nm (Mean ± SD, nm)	50 < *Ø* < 300 nm (Mean ± SD, nm)	*Ø* ≥ 300 nm (Mean ± SD, nm)	PDI
EVs	0–1% (40 ± 10 nm)	99–100% (210 ± 90 nm)	-	0.2
CINVs	-	10–25% (130 ± 40 nm, 65 ± 10 nm)	75–90% (500 ± 220 nm)	0.5
MDNVs (Fr/Th)	3–4% (30 ± 5 nm)	≈70% (140 ± 60 nm)	≈25% (510 ± 170 nm)	0.4
MDNVs (5 min UB)	-	100% (190 ± 130 nm)	-	0.4
MDNVs (15 min UB)	0–6% (30 ± 10 nm)	94–100% (180 ± 90 nm)	-	0.3
MDNVs (30 min UB)	0–0.2% (15 ± 2 nm)	≈90% (180 ± 120 nm)	≈10% (1040 ± 400 nm)	0.5
MDNVs (60 min UB)	-	80% (230 ± 200 nm)	20% (1060 ± 400 nm)	0.5
MDNVs (2 h UB)	3–6% (40 ± 10 nm)	≈70% (160 ± 80 nm)	≈25–30% (1070 ± 400 nm)	0.5
MDNVs (3 h UB)	0–2% (30 ± 10 nm)	98–100% (210 ± 150 nm)	-	0.4
MDNVs (5 min UB + Fr/Th)	0–1% (20 ± 5 nm)	≈75% (150 ± 80 nm)	≈25% (890 ± 380 nm)	0.6
MDNVs (15 min UB + Fr/Th)	-	100% (190 ± 130 nm)	-	0.4
MDNVs (30 min UB + Fr/Th)	2–3% (20 ± 5 nm)	97–98% (230 ± 210 nm)	-	0.6
MDNVs (60 min UB + Fr/Th)	-	50% (90 ± 30 nm)	50% (300 ± 150 nm)	0.5
MDNVs (2 h UB + Fr/Th)	10% (50 ± 10 nm)	50% (150 ± 50 nm)	40% (1870 ± 750 nm)	0.7
MDNVs (3 h UB + Fr/Th)	-	30% (90 ± 30 nm)	70% (810 ± 270 nm)	0.99

MSC—mesenchymal stem cell; EVs—extracellular vesicles; CINVs—cytochalasin-B-inducible nanovesicles; MDNVs—membrane-derived nanovesicles.

## Supplementary Materials

The following are available online at https://www.mdpi.com/2072-666X/10/11/750/s1, Figure S1: The phenotype of human mesenchymal stem cells (MSCs) derived from the endometrial functional layer. Data indicate flow cytometry analysis; Figure S2: MSC differentiation: (a–d) osteo-differentiation, (e–j) chondro-differentiation (intercellular matrix), and (k–l) adipo-differentiation. (a,b) Cells stained with nitrotetrazolium blue; (c,d) calcium staining with alizarin red S; (e,f) staining of acidic glycosaminoglycans with toluidine blue; (g,h) staining of sulfated glycosaminoglycans with alcian blue; (i,j) staining of collagen II with specific antibodies labeled with Сy 3.5 (nucleus stained with DAPI); (k,l) staining of lipid droplets with oil red O. Micrographs in (a,c,e,g,i,k) represent non-induced (control) cells and (b,d,f,h,j,l) show induced cells. Scale bar = 100 μm; Figure S3: DLS data for natural extracellular vesicles (EVs). Peak 2 corresponds to a group of small particles detected in several samples; Figure S4: Aggregates presented in preparations of natural EVs and artificial mimics. TEM analysis. Scale bar = 100 nm; Figure S5: Virus-like particles in human endometrial MCS-derived membrane-derived nanovesicles (MDNVs). Transmission electron micrographs of non-membrane particles that form colonies or are surrounded by fragments of plasma membrane. The MDNVs were generated by 3 h UB followed by Fr/Th. Scale bar = 100 nm; Figure S6: Latex bead doublet discrimination. EVs were immobilized on 4 µm aldehyde/sulfate latex and stained with CD9-FITC conjugate. The R3 region was chosen to detect fluorescence intensity in all further experiments. E5 and E6 regions that corresponded to bead doublet were excluded from the analysis; Figure S7: Ultracentrifugation affects the yield of EVs loaded with FAM-ON. HepG2-derived EVs were loaded with FAM-ON under standard conditions (15 µg EVs were mixed with 5 µM FAM-ON in 200 µL TBS) by sonication, permeabilization with 0.2% saponin, or Fr/Th. EV–FAM-ON complexes were immobilized on 4 µm aldehyde/sulfate latex beads either immediately after loading (light colors) or after re-precipitation by ultracentrifugation (dark colors). Sixty-to-eighty percent of latex beads contained EV–FAM-ON complexes after sonication or Fr/Th and ≈10% after saponin treatment in the absence of ultracentrifugation. This amount was reduced to ≈1% for sonication and Fr/Th and did not change for saponin after vesicle re-precipitation by ultracentrifugation. Fluorescent signal was not detected for sonication and Fr/Th after ultracentrifugation, data that indicate a massive loss of nanovesicles; Figure S8: FAM-ON loading in buffering system and culture mediums. Loading 15 µg EVs, cytochalasin-B-inducible nanovesicles (CINVs), or MDNVs was performed by Fr/Th. FAM-ON concentrations were from 5 to 50 µM for EVs and 50 µM for CINVs and MDNVs. (**a**) EVs. (**b**) CINVs and MDNVs. Rfu level observed in TBS was set at 1 and is shown as the red dashed line.
