# Supplementary material for: Cytochalasin-B-Inducible Nanovesicle Mimics of Natural Extracellular Vesicles That Are Capable of Nucleic Acid Transfer
Source: Micromachines (Basel). 2019 Nov 1;10(11):750. doi: 10.3390/mi10110750 (PMC6915531; doi:10.3390/mi10110750)
Supplement: Supplementary file 1 [file micromachines-10-00750-s001.pdf]

Supporting Information

Cytochalasin-B-Inducible Nanovesicle Mimics of Natural Extracellular Vesicles That Are Capable of Nucleic Acid Transfer

Anastasiya Oshchepkova [1], Alexandra Neumestova [1], Vera Matveeva [1], Lyudmila Artemyeva [1], Ksenia Morozova [2], Elena Kiseleva [2], Marina Zenkova [1,*] and Valentin Vlassov [1]

[1] Institute of Chemical Biology and Fundamental Medicine SB RAS, Novosibirsk 630090, Russia; niboch@niboch.nsc.ru

[2] Institute of Cytology and Genetics SB RAS, Novosibirsk 630090, Russia; icg-adm@bionet.nsc.ru

* Correspondence: marzen@niboch.nsc.ru, +7 (383) 363-51-60

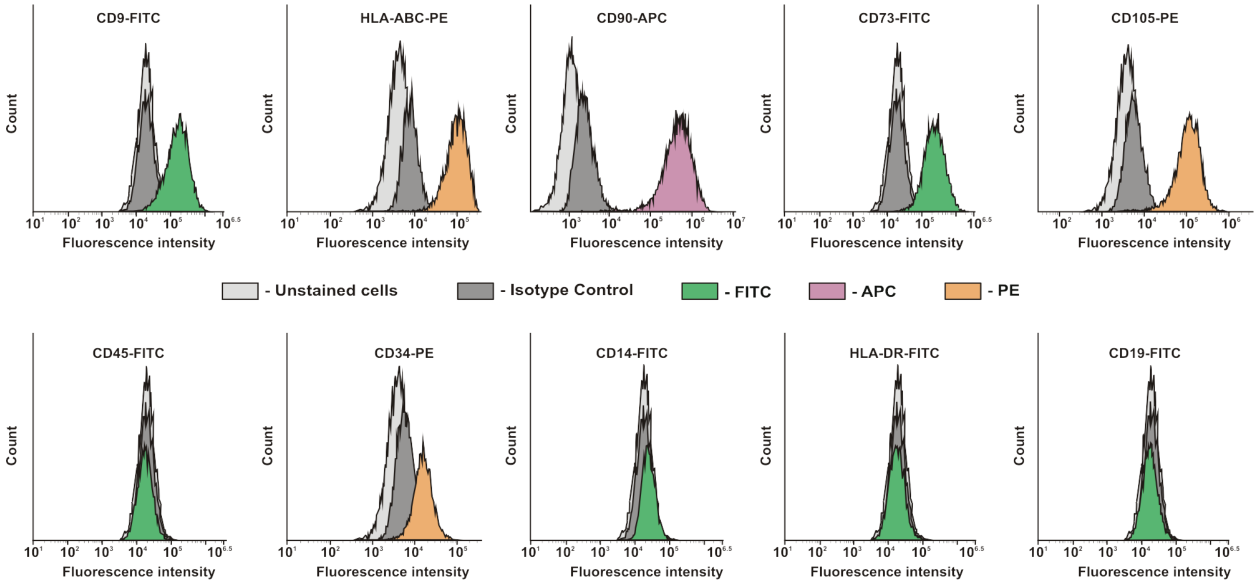

Figure S1. The phenotype of human mesenchymal stem cells (MSCs) derived from the endometrial functional layer. Data indicate flow cytometry analysis.

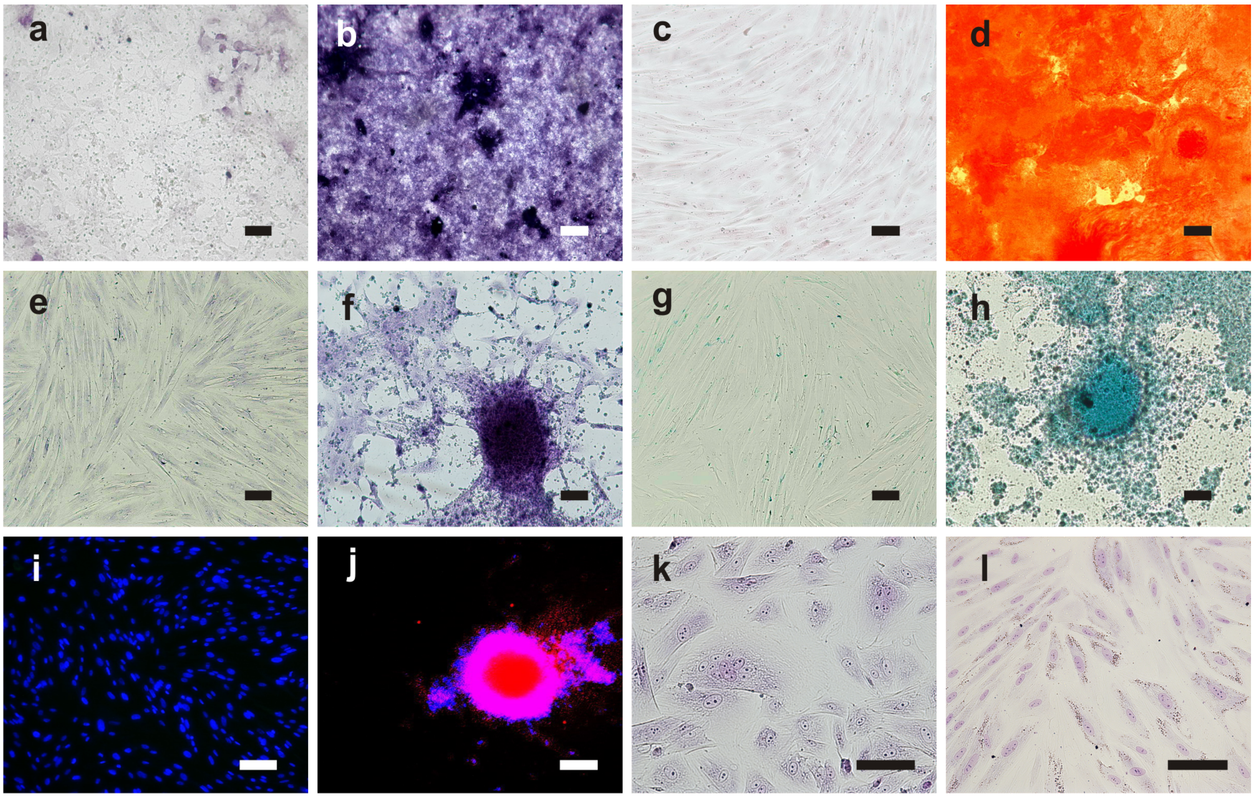

Figure S2. MSC differentiation. (**a**-**d**) Osteo-differentiation, (**e**–**j**) chondro-differentiation (intercellular matrix), and (**k**–**l**) adipo-differentiation. (**a**,**b**) Cells stained with Nitrotetrazolium Blue; (**c**,**d**) calcium staining with Alizarin Red S; (**e**,**f**) staining of acidic glycosaminoglycans with Toluidine Blue; (**g**,**h**) staining of sulfated glycosaminoglycans with Alcian Blue; (**i**,**j**) staining of collagen II with specific antibodies labeled with Cy 3.5 (nucleus stained with DAPI); (**k**,**l**) staining of lipid droplets with Oil Red O. Micrographs in (**a**,**c**,**e**,**g**,**i**,**k**) represent non-induced (control) cells and (**b**,**d**,**f**,**h**,**j**,**l**) show induced cells. Scale bar = 100 µm.

			Size (d.nm):	% Intensity:	St Dev (d.nm):
Z-Average (d.nm):	179.3	Peak 1:	212.7	99.2	93.15
PdI:	0.204	Peak 2:	38.68	0.8	6.840
Intercept:	0.914	Peak 3:	0.000	0.0	0.000
Result quality:	Good				

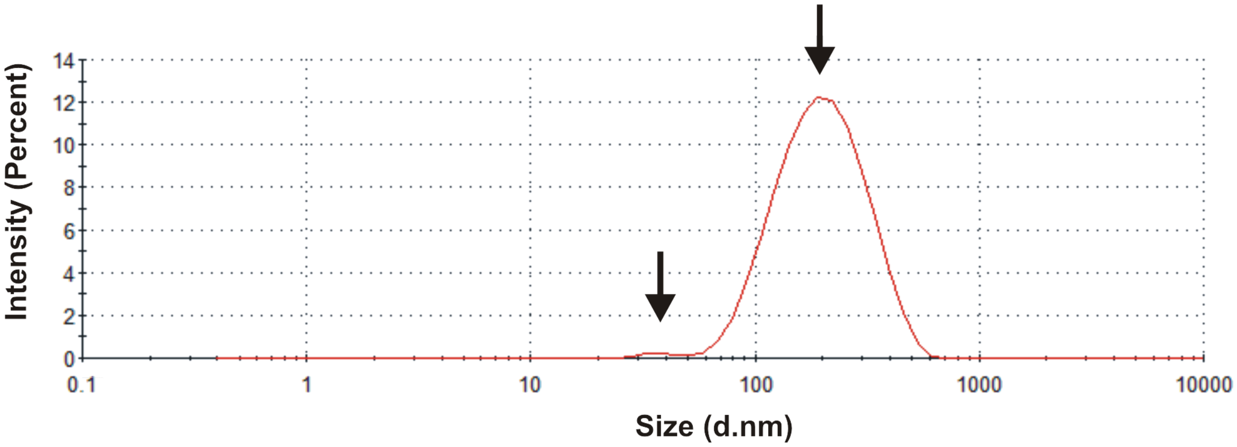

Figure S3. DLS data for natural extracellular vesicles (EVs). Peak 2 corresponds to a group of small particles detected in several samples.

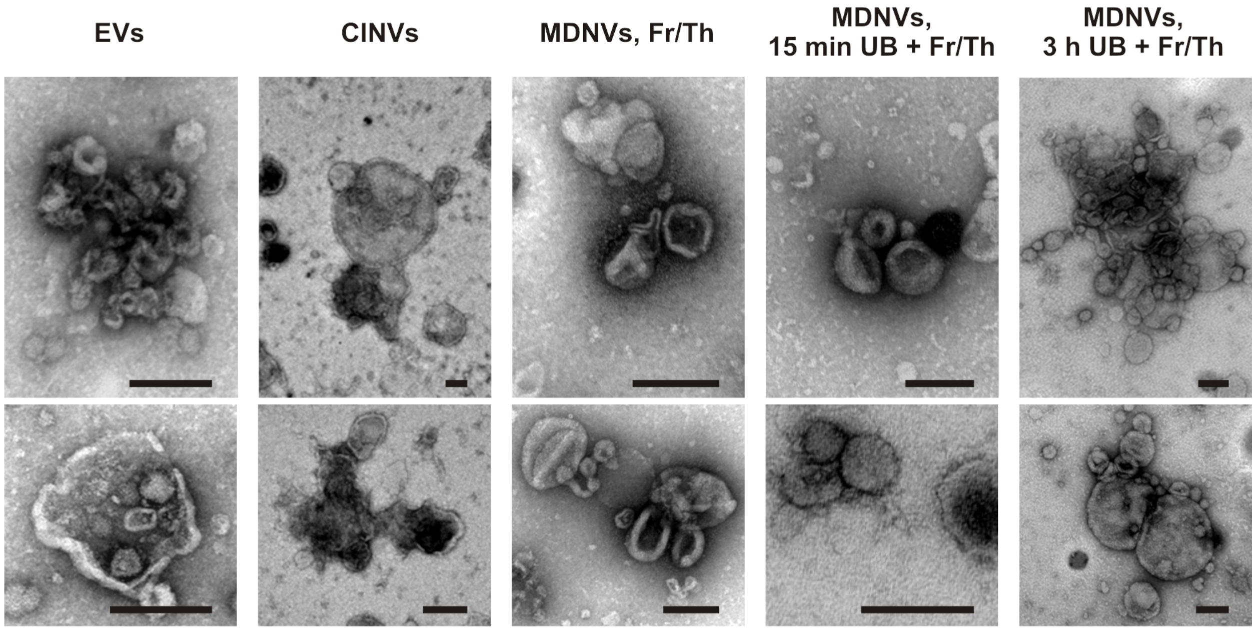

Figure S4. Aggregates presented in preparations of natural EVs and artificial mimics. TEM analysis. Scale bar = 100 nm.

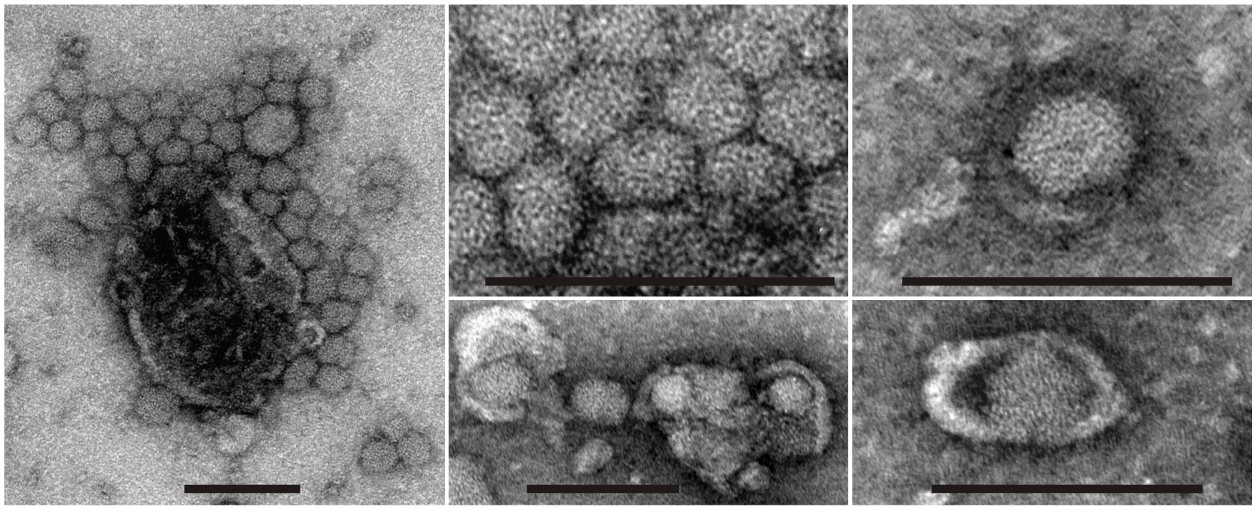

Figure S5. Virus-like particles in human endometrial MCS-derived membrane-derived nanovesicles (MDNVs). Transmission electron micrographs of non-membrane particles that form colonies or are surrounded by fragments of plasma membrane. The MDNVs were generated by 3 h UB followed by Fr/Th. Scale bar = 100 nm.

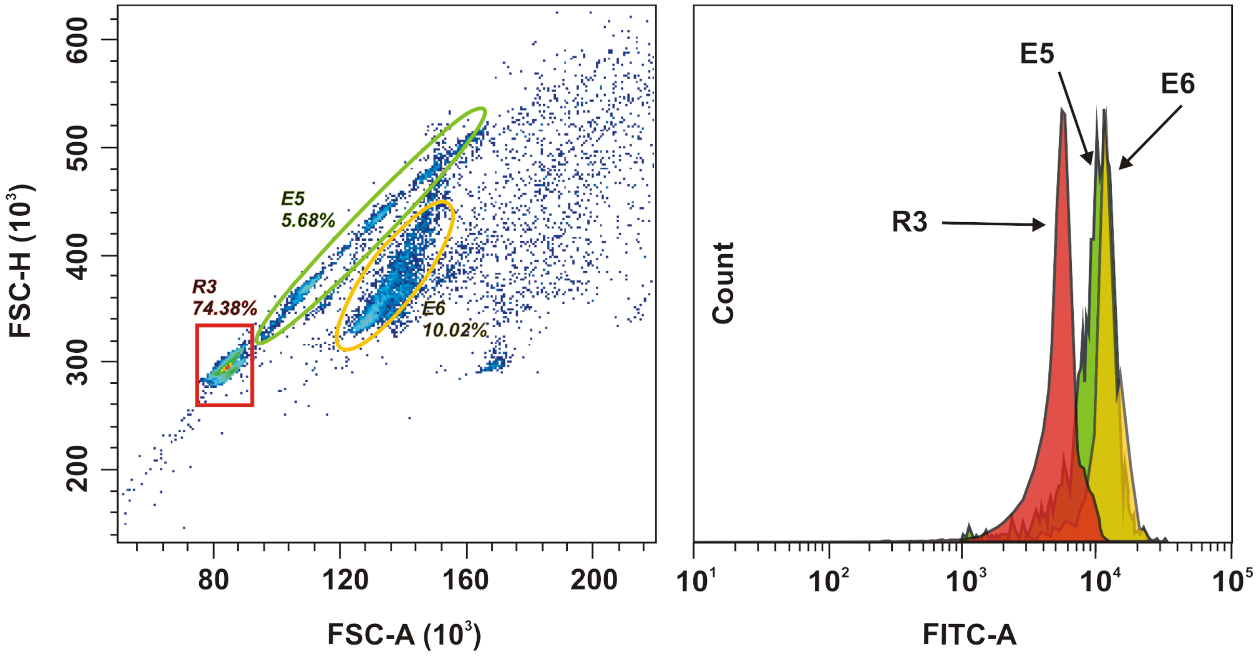

Gate	Count	% All	Mean X	Mean Y	CV X	CV Y
All	30 158	100%	101.312	322.939	37.31%	21.09%
R3	22 432	74.38%	84.414	295.205	2.19%	1.76%
E5	1 714	5.68%	123.509	408.081	14.28%	12.33%
E6	3 023	10.02%	135.368	366.358	4.83%	7.71%

Gate	Count	%	Mean X	CV X
R3	22 448	98.82%	5.392	35.90%
E5	1 838	98.91%	10.273	35.28%
E6	3 518	99.86%	12.614	28.72%

Figure S6. Latex bead doublet discrimination. EVs were immobilized on 4 µm aldehyde/sulfate latex and stained with CD9-FITC conjugate. The R3 region was chosen to detect fluorescence intensity in all further experiments. E5 and E6 regions that corresponded to bead doublet were excluded from the analysis.

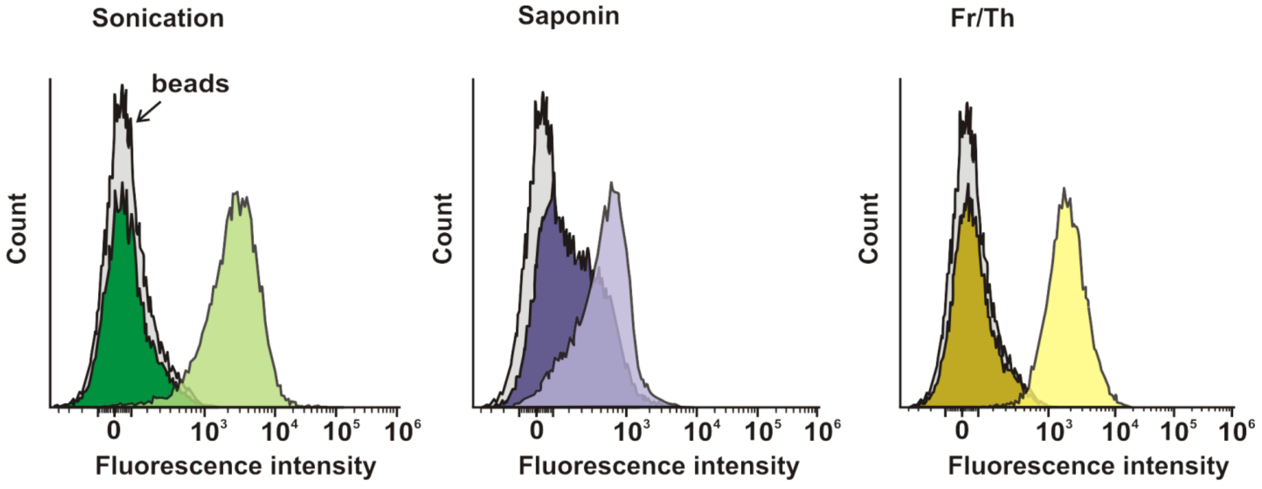

Figure S7. Ultracentrifugation affects the yield of EVs loaded with FAM-ON. HepG2-derived EVs were loaded with FAM-ON under standard conditions (15 µg EVs were mixed with 5 µM FAM-ON in 200 µL TBS) by sonication, permeabilization with 0.2% saponin, or Fr/Th. EV-FAM-ON complexes were immobilized on 4 µm aldehyde/sulfate latex beads either immediately after loading (light colors) or after re-precipitation by ultracentrifugation (dark colors). Sixty-to-eighty percent of latex beads contained EV-FAM-ON complexes after sonication or Fr/Th and ≈10% after saponin treatment in the absence of ultracentrifugation. This amount was reduced to ≈1% for sonication and Fr/Th and did not change for saponin after vesicle re-precipitation by ultracentrifugation. Fluorescent signal was not detected for sonication and Fr/Th after ultracentrifugation, data that indicate a massive loss of nanovesicles.

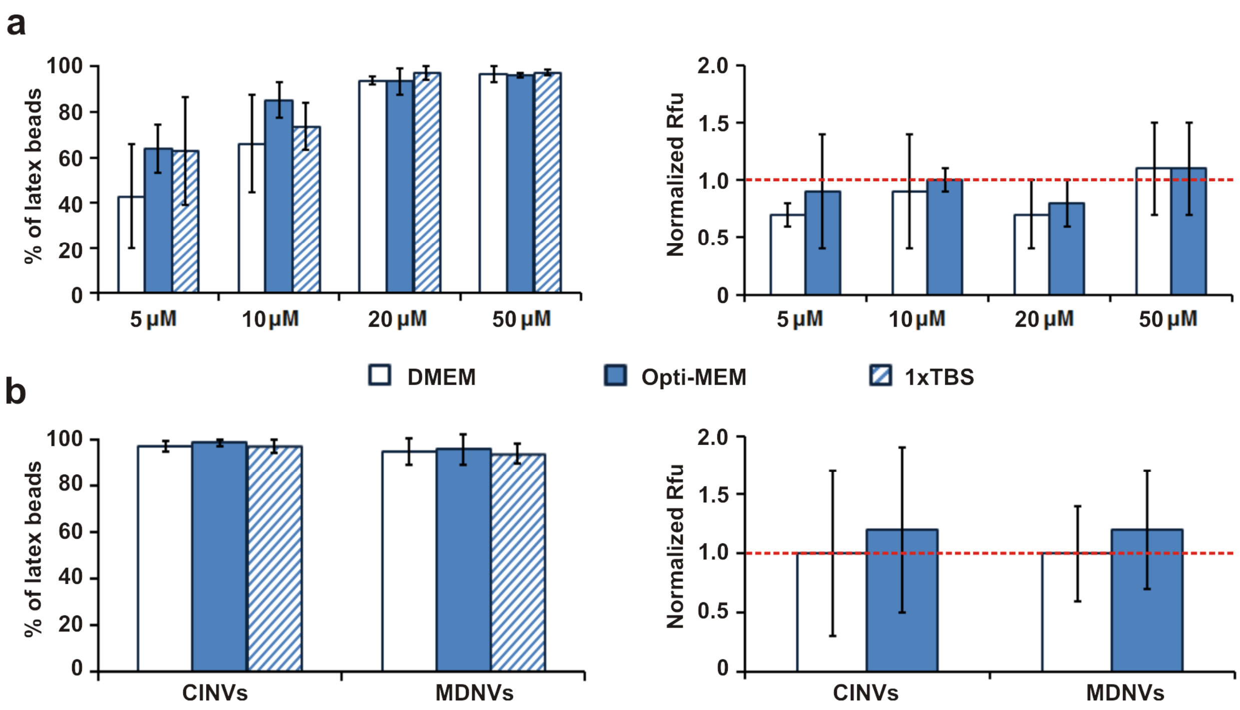

Figure S8. FAM-ON loading in buffering system and culture mediums. Loading 15 µg EVs, cytochalasin-B-inducible nanovesicles (CINVs), or MDNVs was performed by Fr/Th. FAM-ON concentrations were from 5 to 50 µM for EVs and 50 µM for CINVs and MDNVs. (**a**) EVs. (**b**) CINVs and MDNVs. Rfu level observed in TBS was set at 1 and is shown as the red dashed line.
